# Editorial: Neuroscience of human attachment volume II

**DOI:** 10.3389/fnhum.2022.1018528

**Published:** 2022-08-31

**Authors:** Anna Buchheim, Carol George, Harald Gündel

**Affiliations:** ^1^Institute of Psychology, University of Innsbruck, Innsbruck, Austria; ^2^Department of Psychology, Mills College, Oakland, CA, United States; ^3^Department of Psychosomatic Medicine and Psychotherapy, Ulm University, Ulm, Germany

**Keywords:** attachment, attachment measures, human neuroscience, fMRI, psychophysiology, endocrine markers, genetic markers

The Research Topic “Neuroscience of human attachment volume II” includes state of the art papers representing an innovative spectrum of current approaches to investigate biologically based systems associated with significant relationships. The included studies apply various neurobiological measures to examine neural signatures of attachment in clinical and non-clinical samples *over life span*.

The first part of the volume deals with *intergenerational issues of attachment and trauma in children and parents*. Most transgenerational studies examined how parents' attachment representations link to their children's attachment development (e.g., Madigan et al., 2019; van IJzendoorn and Bakermans-Kranenburg, 2019). However, no research has investigated how parents' attachment is associated to the children's brain development, and no study has implemented a prospective *and* trans-disciplinary study approach to identifying protective factors helping to disrupt impaired intergenerational effects.

The paper by Fitter et al. focused on how healthy mothers' attachment representations relate to the children's brain development. Mothers' greater secure base script knowledge, but not attachment style, was associated with smaller left amygdala volume in early childhood. These pilot findings with 3–8-year old children underline a possible role of parents' secure base script knowledge specifically in predicting amygdala development in early childhood and suggest that maternal representations may be linked to the development of children's neural circuitry related to stress regulation.

The paper by Buchheim et al. examined in the “TRANS-GEN” project consequences of maternal childhood maltreatment (CM) and their effects of resilience factors on child attachment and stress-regulatory development. The authors focused on the role of the attachment and social support system and biological stress susceptibility in mother-child dyads, as these systems are known to affect the consequences of CM to the next generation. The authors confirmed that maternal CM experiences were significantly associated with unresolved attachment, higher perceived stress and more psychological symptoms. These negative effects of CM were buffered by social support. Maternal organized attachment was significantly associated with organized child attachment demonstrating an additional protective factor. Concerning the child's biological stress susceptibility, the rs2254298 genotype of the OXTR gene moderated the stress response of children from mothers with CM. In sum the authors replicated and extended existing CM and attachment models (van IJzendoorn and Bakermans-Kranenburg, 2019). Especially the consideration of genetic risk and resilience factors could open the gate toward personalized treatment approaches for parents and their children.

Dealing with adverse childhood and trauma in a secure relationship is crucial to prevent pathological adjustment. From this perspective the paper by Hug et al. introduces a study protocol within the project “MuKi” to examine the effects of developmental trauma disorder on reward expectancy and processing in children aged 8–12 years, testing the hypothesis that children with multiple complex traumas exhibit altered reward processing as a result of prior disappointing reward experiences. The authors put in promising that if the results of the planned behavioral study are working, the MID task will be used in a future study to elucidate the relationship between trauma developmental disorder and neurobiological processes in middle childhood.

The second part of the volume includes focuses on *physiological and genetic markers of attachment in middle childhood and adolescence*.

Since most previous studies examining gene-environment effects on self-regulation focused only on outcomes of early childhood or adulthood the paper by Zimmermann and Spangler aimed to investigate longitudinal effects during middle childhood and adolescence. In a longitudinal follow-up the authors studied the effects of differences in the DRD4 tandem repeat polymorphisms and two domains of early maternal caregiving quality on children's personality development at age six and age 12 and on problem behavior at ages six and seven. The results highlight that especially early effective maternal regulation of infants' distress can help children with the DRD4 7+ variant to develop adaptive self-regulation in middle childhood and adolescence and suggests their importance on personality development over life span.

Previous work demonstrates that little is known about differences in attachment classifications and physiological stress response in adolescent age groups (Gander and Buchheim, 2015). The paper by Gander et al. examined heart rate (HR) and heart rate variability (HRV) during an attachment interview in adolescents. Secure adolescents showed a higher HRV from baseline to the attachment interview compared to insecure ones, indicating that secure adolescents were more capable of dealing with attachment-related distress which is represented in higher HRV. Moreover, HRV is increasingly recognized as a marker of feeling safe and connected in social environments (Bryant and Hutanamon, 2018) and thus these results might have important implications for psychotherapy research.

One robust finding in clinical attachment research is the relationship between unresolved attachment and psychopathology, especially in Borderline Personality Disorders (BPD) (Buchheim and Diamond, 2018). The paper by Bernheim et al. examined attachment representations of BPD patients and healthy controls and administered an fMRI-adapted attachment paradigm to investigate neural correlates of attachment. As expected, unresolved attachment was predominant in BPD and patients showed increased fMRI-activation in brain areas associated with fear, pain, and hyperarousal than controls when presented with personalized attachment-relevant alone stimuli. The results replicated previous results (Buchheim et al., 2008) that illustrated again the relevance of aloneness and feelings of abandonment for BPD (Gunderson, 1996; Schmahl et al., 2003).

We invited authors for the first (Buchheim et al., 2017) and this second Research Topic in Frontiers in Human Neuroscience to submit original research or reviews that addressed topics in the neurobiological domain related to any aspect of attachment that would highlight promising avenues for basic research or the translation of attachment studies into the clinical setting. The authors were reporting on different age groups and used various methodological approaches to respond to this topic. As a result, we again achieved an exciting interdisciplinary synthesis of existing knowledge and new perspectives on the human neuroscience of attachment that demonstrates the tremendous development in this field. As a next step, we would like to encourage attachment researchers to use transdiagnostic approaches and evaluate the effectiveness of preventive programs and attachment related interventions with neurobiological, endocrine or genetic parameters.

## Author contributions

AB has written the editorial and the co-authors have approved and agreed.

## Conflict of interest

The authors declare that the research was conducted in the absence of any commercial or financial relationships that could be construed as a potential conflict of interest.

## Publisher's note

All claims expressed in this article are solely those of the authors and do not necessarily represent those of their affiliated organizations, or those of the publisher, the editors and the reviewers. Any product that may be evaluated in this article, or claim that may be made by its manufacturer, is not guaranteed or endorsed by the publisher.
